# Embolization of a Large Right-Coronary-Artery-to-Left-Atrium Fistula in a Three-Year-Old Child: A Case Report

**DOI:** 10.3390/jcdd11100298

**Published:** 2024-09-25

**Authors:** Stasa Krasic, Gianfranco Butera, Vesna Topic, Vladislav Vukomanovic

**Affiliations:** 1 Cardiology Department, Mother and Child Health Institute of Serbia, R. Dakica St. 6-8, 11070 Belgrade, Serbia; 2 Faculty of Medicine, University of Belgrade, 11000 Belgrade, Serbia; 3 Cardiology, Cardiac Surgery and Heart Lung Transplantation, ERN GUARD HEART, Bambino Gesù Hospital and Research Institute, IRCCS, 00165 Rome, Italy; 4 Radiology Department, Mother and Child Health Institute of Serbia, R. Dakica St. 6-8, 11070 Belgrade, Serbia

**Keywords:** coronary artery fistulas, right coronary artery, Amplatzer vascular plug 4, children

## Abstract

Objectives: Coronary artery fistulas (CAFs) are rare congenital anomalies with an occurrence rate of 0.002–0.3%. The right coronary artery (RCA) is reportedly the most common site of origin of CAFs, but fistulas draining to the left atrium (LA) are rare. We presented a three-year-old boy with a symptomatic congenital RCA-to-LA fistula, which was successfully percutaneously occluded with an Amplatzer vascular plug 4 (AVP4). Case report: The diagnosis was made by echocardiography when he was two months old. During the follow-up period of 2 years, a progressive dilatation of the RCA and enlargement of the left ventricle was detected, so treatment for congestive heart failure was initiated. At the age of three, the patient presented with a history of occasional mild central chest pain and discomfort and mild dyspnea on exertion. On a 24 h ECG Holter monitor, the depression of ST segments was registered. CT angiography highlighted a large type B RCA fistula to the LA, which extended along the atrioventricular sulcus. The proximal RCA diameter was 7 mm. The fistula was tortuous, with segmental narrowing and three curves. Cardiac catheterization was performed across the right femoral artery on the three-year-old boy (body weight: 13 kg). Across the 4F Judkins right guiding catheter, an AVP4 of 5 mm was placed in the distal part of the CAF connected with the delivery cable. After 15 min, ECG changes were not registered, so the device was released. Immediate post-deployment angiography demonstrated complete CAF occlusion, with satisfying flow in the distal coronary artery. The patient was discharged after four days. In the short-term follow-up period, the boy was symptom-free. Conclusions: In our experience, given the existence of the left-to-left shunt and the more pronounced exercise-induced coronary steal phenomenon that occurs in medium-sized and large CAFs, occlusion is necessary to prevent the further progression of clinical signs and symptoms.

## 1. Introduction

The incidence of coronary artery (CA) anomalies is 0.2 to 1.2% of the population, and coronary artery fistulas (CAF) comprise 48.7% of all congenital coronary anomalies [1]. The prevalence is 0.1% to 0.2% in the general population, while CAFs are present in 11% of patients younger than 20 years [1,2]. Failure to achieve the obliteration of the embryonic sinusoids that perfuse the primitive myocardium leads to a fistulous connection between the CA and cardiac chambers (coronary cameral fistula). The right coronary artery (RCA) is reportedly the most common site of origin of CAFs, accounting for 50–60% of cases, but fistulas draining to the left atrium (LA) are rare (5–6%) [1,3]. Besides those that are congenital, CAFs can be acquired (cardiac trauma, procedures (coronary stent placement, coronary artery bypass grafting, chest irradiation, etc.) or caused by cardiac disease (myocardial infarction and coronary vasculitis)) [1,2,3].

Coronary cameral fistula is the most common type of coronary artery fistula in children, accounting for 75–100% of cases. The most common draining site is the right ventricle, followed by the left ventricle, and, less frequently, the right or left atrium [4].

CAFs can be classified as small, medium, or large if the fistula diameter is <1, 1 to 2, or >2 times larger than the largest diameter of the non-feeding coronary artery, respectively [2]. Despite this classification, Sakakibara et al. suggested angiographic classification: Type A, proximal type, where the dilatation of the proximal coronary segment before the fistula origin and the distal end is average, and Type B, distal type, where the CA is dilated over its entire length, terminating as a fistula flowing mainly into the right side of the heart (end-artery type) and the proximal coronary segment may have regular branches [1,3]. This classification system helps to categorize CAFs based on their anatomical features and can guide treatment decisions.

In sizable fistulas, marked shunting and progressive dilatation of the fistula are registered. The improper flow of oxygen-rich blood can overload the heart chambers, leading to an increased volume load and potential heart dysfunction over time. Moreover, the abnormal flow dynamics can predispose children to ischemic events, especially if there is a significant steal phenomenon [1,2]. Approximately 19–63% of patients begin to show symptoms by the age of 18 years [3]. Due to progressive proximal CA dilatation over the years, medium-size fistulas should be closed early before further growth, as the closure of larger fistulas is associated with an increased risk for myocardial infarction (MI) [2]. Additionally, large CAFs might cause pulmonary hypertension, lead to high-output failure, compress adjacent structures, or cause endocarditis or in situ thrombus [5].

We present a three-year-old boy with a rare and symptomatic RCA-to-LA fistula, which was successfully percutaneously occluded with an Amplatzer vascular plug 4.

## 2. Case Report

**Figure 1:**
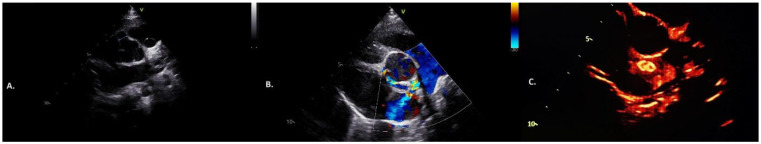
Transthoracic echocardiography revealed the dilated right coronary artery (6 mm diameter) (**A**) and the drainage place of the right-coronary-artery fistula (**B**). Postprocedural echocardiography revealed a well-positioned AVP4 (**C**).

**Figure 2:**
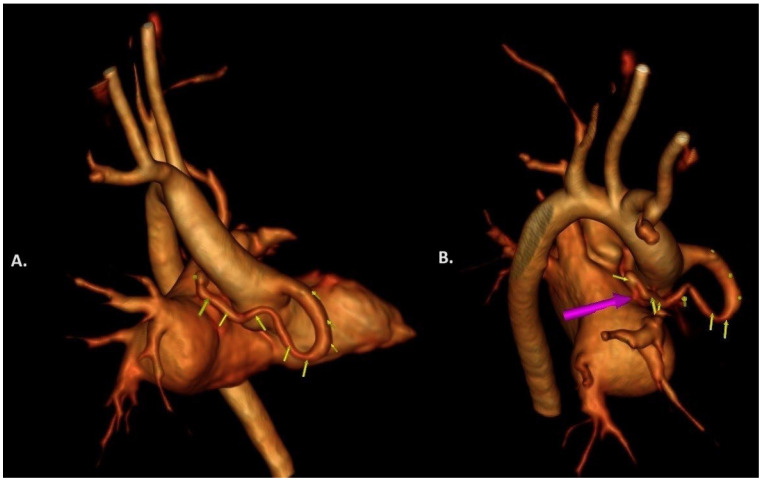
CT angiography 3D reconstruction highlighted a tortuous type B right coronary artery fistula (**A**) to the left atrium (yellow arrows) and the drainage place (pink arrow) (**B**).

In a two-month-old boy, a continuous heart murmur was registered. Echocardiography examination revealed a right coronary–cameral fistula (Figure 1A,B). The origin of the right coronary artery (RCA) was dilated, and fistula drainage was detected in the left atrium (LA). During a follow-up period of 2 years, a progressive dilatation of the RCA (6–7 mm in diameter) and enlargement of the left ventricle were detected (end-diastolic diameter Z score: +2.5), so treatment for congestive heart failure (furosemide, spironolactone, and bisoprolol) was initiated. At the age of three, the patient presented with a history of occasional mild central chest pain and discomfort and mild dyspnea on exertion. On a 24 h ECG Holter, the depression of ST segments to 0.41 mV was registered at maximal heart rate. ECG-gated multidetector computed tomography (MD-CT) angiography with 3-dimensional volume-rendered reconstruction was performed to evaluate the CAF. The CT angiography highlighted a large type B RCA fistula to the LA (‘Sakakibara’ classification) (Figure 2), which extended along the atrioventricular sulcus. The proximal RCA diameter was 7 mm. The fistula was tortuous, with segmental narrowing and three curves. The diameters were 3.5–4.6 mm, while the drainage place of the CAF in the LA was 2.1 mm. The LCA diameter was 2.2 mm.

The patient’s medical record was presented to our heart team, and we decided to perform transcatheter occlusion. 

**Figure 3:**
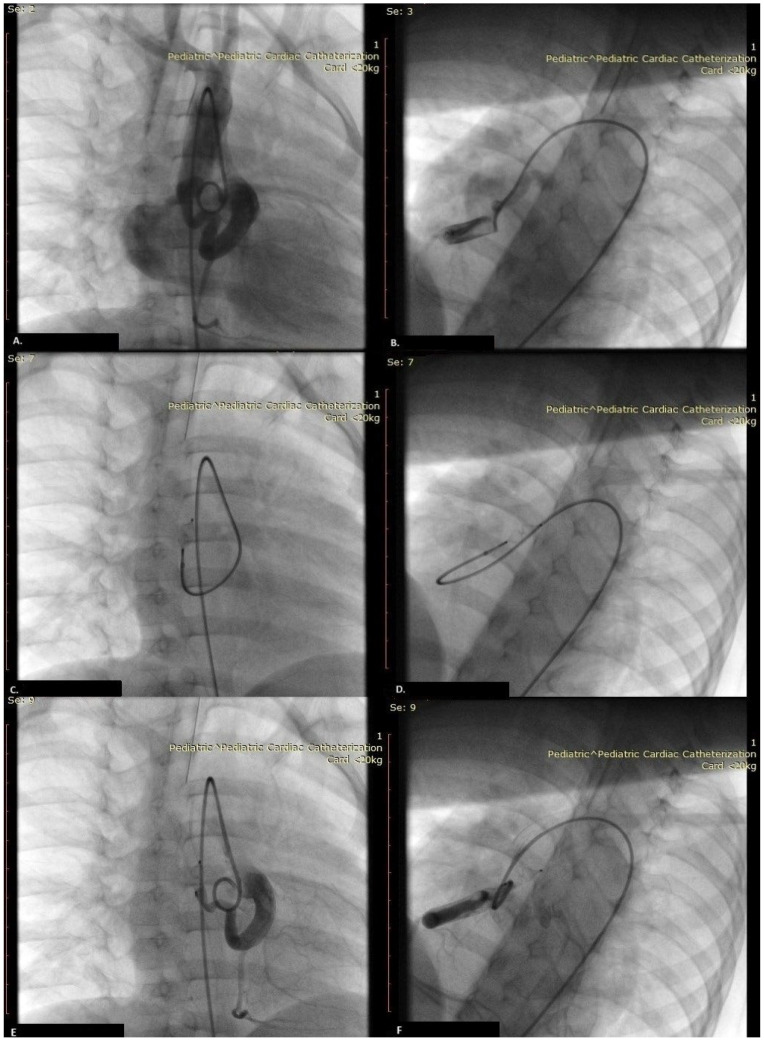
Aortography in the RAO 30′ and LAO 30′ cranial 20′ positions was identified in the distal part of the right coronary artery (right acute marginal branch) and coronary artery fistula (CAF) (**A**,**B**). An AVP4 connected to the delivery cable was placed in the distal CAF part (**C**,**D**). Coronarography after AVP4 deployment revealed complete CAF occlusion, with satisfying flow in the distal coronary artery (**E**,**F**).

Cardiac catheterization was performed across the right femoral artery (retrograde transarterial access) on the three-year-old boy (body weight 13 kg). One hundred U/kg heparin was administered. Firstly, aortography was performed in the RAO 30′ and LAO 30′ cranial 20′ positions to identify RCA and coronary artery fistula (CAF). Selective coronarography revealed the origin of the distal RCA and large tortuous fistula (3.5–4.5 mm in diameter) (Figure 3A,B). The last RCA branch was the right marginal artery. TERUMO Glide Technology™ 0.035-inch hydrophilic angled wire was easily positioned from the aorta into the LA across the CAF. At the same time, a 0.014-inch coronary guidewire was unstable and could not navigate either the Progreat microcatheter or 4F guide catheter in this extremely tortuous fistula. Across the 4F Judkins right guiding catheter, an Amplatzer Vascular Plug 4 (AVP4) (St. Jude Medical) of 5 mm was placed in the distal part of the CAF connected with the delivery cable (Figure 3C,D). After 15 min (device occlusion test), ECG changes were not registered, so the device was released. Immediate post-deployment angiography demonstrated complete CAF occlusion with CAF thrombosis, satisfying flow in the distal coronary artery with better right marginal artery opacification (Figure 3E,F, Supplementary Video S1). We started with aspirin on the day of the procedure at a dose of 5 mg/kg. Control echocardiography revealed a well-positioned AVP4 without residual flow (Figure 1C). The patient was discharged after four days. 

During the short-term follow-up period, the boy was symptom-free. Cardiac troponin I was in the referent range three months after the procedure. Serial echocardiography examinations revealed a well-positioned AVP4 without residual flow with both chambers’ normal systolic and diastolic function. The LV EDD Z score decreased from +2.5 to +0.8, while the RCA diameter decreased from 7 mm to 4 mm. 

## 3. Discussion

We successfully performed a large type B RCA-LA CAF transcatheter occlusion on the three-year-old boy. According to the literature data, congenital RCA-LA fistulas are rare; the first was described in 1967 [6], and only sporadic cases have been reported in adults [3,7,8,9]. None of the CAFs were percutaneously occluded in these cases. In our hospital, four coronary–cameral fistulas have been closed, two surgically and two percutaneously [10].

The left atrium drains CAFs to mimic mitral regurgitation, while fistulas drain into the left ventricle to mimic aortic regurgitation [5]. The left-to-left shunt in our patient could explain the dilatation of the left atrium and ventricle. The ST segment depression on 24 h ECG Holter monitoring at maximal heart rate resulted from myocardial tissue bypass during increased demand. 

Reidy et al. first performed the transcatheter closure of a CAF in 1983 using a detachable balloon technique [11]; since then, the transcatheter closure of fistulas has been widely used as an effective and safe treatment. According to the American College of Cardiology/American Heart Association (ACC/AHA) guidelines, transcatheter occlusion is indicated for patients with symptomatic CAF (Class I, Level of Evidence: B), while transcatheter occlusion is reasonable in the management of symptom-free patients with a medium or large CAF (Class IIa; Level of Evidence: C) [12]. The updated 2018 ACC/AHA guidelines emphasize the importance of a heart team approach to evaluate the appropriateness and feasibility of CAF closure at centres with expertise in percutaneous and surgical closure techniques [13]. Although type A CAF is ideal for transcatheter closure, our heart team decided on percutaneous occlusion. At the same time, the patient had symptoms, progressive RCA dilatation, a significant left-to-left shunt (dilatation of the left ventricle) and ST depression on 24 h ECG, and, on the other hand, the proximal RCA diameter was <10 mm. If the CA diameter is >10 mm, then patients should undergo surgery [2]. 

Although AV loop and transvenous access are preferable for distal CAF occlusion [2], we use a transarterial approach due to drainage in the LA. 

The Amplatzer vascular plug 4 is a self-expanding, bi-lobar plug. We decided on occlusion with the AVP4 due to its easy deliverability through tortuous segments and compatibility with a 0.038-inch-inner-diameter catheter [14]. As our patient was three years old and 13 kg, we wanted to use the smallest guide catheter possible to reach the CAF and access the femoral artery to avoid MI and femoral thrombosis. Additionally, since it is detachable, we monitored ECG changes for 15 min before it was released. 

The larger the CAF with a more significant shunt, the higher the risk for coronary events, especially in younger patients with a tortuous CAF [15]. The Achilles’ heel of closing a CAF originating from distal coronary segments is MI due to stagnant flow after closure, but we did not have any complications [2]. We placed the device as distally as possible, >1 cm away from the origin of the fistula, to reduce the risk of device prolapse and thrombus propagation. 

Sang et al. used the quantitative flow ratio (QFR) to interrogate the impact of CAF occlusion and residual shunt on blood flow in donor vessels. They concluded that medium CAF occlusion enhances the functionality of donor vessels, while small CAFs exert no influence on donor blood flow [16]. On the other hand, Saighi Bouaouina et al. and Huang et al. reported an improvement in the FFR value from 0.80 and 0.73 under maximal hyperemia to 0.95 and 0.92, respectively, after the CAF closure [17,18]. This may be the explanation for the better opacification of the right marginal coronary artery after the occlusion of the CAF in our patient. 

Our patient had optimal post-closure coronary remodelling [15]. At the same time, post-procedural coronagraphy relieved CAF thrombosis and improved opacification of the right marginal artery, and serial echocardiographic examinations showed a subsequent decrease in RCA diameter.

After the procedure, we started aspirin. Although our patient has a large distal-type CAF, he will use an antiagregation drug for 12 months.

We present a 3-year-old child with a congenital RCA-LA CAF that we successfully occluded using an AVP4. CAFs represent a unique subset of congenital heart anomalies that can have profound implications for pediatric patients. With advancements in imaging and interventional techniques, embolization has become a cornerstone in managing these cases. Through effective CAF occlusion, symptoms can be alleviated and the risk of long-term complications can be reduced in affected children. Ongoing research and clinical experience will continue to refine these approaches, ultimately enhancing outcomes for this vulnerable population. Ensuring timely diagnosis and appropriate intervention will be critical in managing coronary cameral fistulas in the pediatric cohort, paving the way for improved quality of life and heart health.

## Data Availability

The datasets generated and analyzed for this study can be requested from the corresponding author.

## Supplementary Materials

The following supporting information can be downloaded at: https://www.mdpi.com/article/10.3390/jcdd11100298/s1, Supplementary Video S1. Percutaneous embolization of right-coronary-artery-to-left-atrium fistula in three-year-old boy.
